# Substantial increase in perfluorocarbons CF_4_ (PFC-14) and C_2_F_6_ (PFC-116) emissions in China

**DOI:** 10.1073/pnas.2400168121

**Published:** 2024-07-15

**Authors:** Minde An, Ronald G. Prinn, Luke M. Western, Bo Yao, Xingchen Zhao, Jooil Kim, Jens Mühle, Wenxue Chi, Christina M. Harth, Jianxin Hu, Anita L. Ganesan, Matthew Rigby

**Affiliations:** ^a^Center for Global Change Science, Massachusetts Institute of Technology, Cambridge, MA 02139; ^b^College of Environmental Sciences and Engineering, Peking University, Beijing 100871, China; ^c^School of Chemistry, University of Bristol, Bristol BS8 1TS, United Kingdom; ^d^Department of Atmospheric and Oceanic Sciences & Institute of Atmospheric Sciences, Fudan University, Shanghai 200438, China; ^e^Meteorological Observation Centre of China Meteorological Administration, Beijing 100081, China; ^f^Shanghai Key Laboratory of Ocean-land-atmosphere Boundary Dynamics and Climate Change, Shanghai 200438, China; ^g^Scripps Institution of Oceanography, University of California San Diego, La Jolla, CA 92093; ^h^School of Geographical Sciences, University of Bristol, Bristol BS8 1SS, United Kingdom

**Keywords:** perfluorocarbons, greenhouse gas, inverse modeling, emissions, climate change

## Abstract

The perfluorocarbons tetrafluoromethane (CF_4_, PFC-14) and hexafluoroethane (C_2_F_6_, PFC-116) are potent greenhouse gases with near-permanent atmospheric lifetimes relative to human timescales and global warming potentials thousands of times that of CO_2_. Using long-term atmospheric observations from a Chinese network and an inverse modeling approach (top–down method), we determined that CF_4_ emissions in China increased from 4.7 (4.2-5.0, 68% uncertainty interval) Gg y^−1^ in 2012 to 8.3 (7.7-8.9) Gg y^−1^ in 2021, and C_2_F_6_ emissions in China increased from 0.74 (0.66-0.80) Gg y^−1^ in 2011 to 1.32 (1.24-1.40) Gg y^−1^ in 2021, both increasing by approximately 78%. Combined emissions of CF_4_ and C_2_F_6_ in China reached 78 Mt CO_2_-eq in 2021. The absolute increase in emissions of each substance in China between 2011-2012 and 2017-2020 was similar to (for CF_4_), or greater than (for C_2_F_6_), the respective absolute increase in global emissions over the same period. Substantial CF_4_ and C_2_F_6_ emissions were identified in the less-populated western regions of China, probably due to emissions from the expanding aluminum industry in these resource-intensive regions. It is likely that the aluminum industry dominates CF_4_ emissions in China, while the aluminum and semiconductor industries both contribute to C_2_F_6_ emissions. Based on atmospheric observations, this study validates the emission magnitudes reported in national bottom–up inventories and provides insights into detailed spatial distributions and emission sources beyond what is reported in national bottom–up inventories.

## Significance

We investigate the emissions of two potent greenhouse gases, perfluorocarbons tetrafluoromethane (CF_4_, PFC-14) and hexafluoroethane (C_2_F_6_, PFC-116), in China. Based on atmospheric observations within China, we report substantial increases in CF_4_ and C_2_F_6_ emissions in China over the last decade. These increases in national emissions are sufficient to explain the entire increases in global emissions over the same period. We suggest that substantial CF_4_ and C_2_F_6_ emissions could be due to by-product emissions from the aluminum industry in the less populated and less economically developed western regions in China. The findings highlight the importance of mitigating CF_4_ and C_2_F_6_ emissions in China and provide guidance for directing mitigation strategies toward specific regions and/or industries.

The perfluorocarbons (PFCs) are potent greenhouse gases which previously had their emissions regulated under the Kyoto Protocol and are currently included in the nonbinding Paris Agreement (1–3). Tetrafluoromethane (CF_4_, PFC-14) and hexafluoroethane (C_2_F_6_, PFC-116), with atmospheric lifetimes of 50,000 and 10,000 y, respectively, are the two most abundant PFCs in the atmosphere (4–6). They have large global warming potentials over a 100-y time horizon (GWP_100_), of 7,380 for CF_4_ and 12,400 for C_2_F_6_ (6). Due to their exceptionally long atmospheric lifetimes, emissions of CF_4_ and C_2_F_6_ alter the global radiative budget essentially permanently compared to human time scales. Substantial by-product emissions of CF_4_ and C_2_F_6_ are created during metal smelting (mainly aluminum) due to anode effects (see refs. 7–10 and references therein), causing the majority of their historic anthropogenic emissions. CF_4_ and C_2_F_6_ have also been used extensively as plasma etching gases in the semiconductor and flat-panel display industries, where release to the atmosphere can occur if the unused gas is not recovered or properly destroyed (7–10).

Measurements of CF_4_ and C_2_F_6_ (from air trapped in ice cores, firn air, archived air, and in situ) have shown preindustrial CF_4_ abundances of ~34.1 ppt (19th century) due to a very small natural source, but less than 0.002 ppt of C_2_F_6_ (11). Global background mole fractions of both CF_4_ and C_2_F_6_ have been increasing due to anthropogenic activities since the early 1900s, with a rapid increase around 1940 due to the aluminum demand during World War II (11, 12). The global average mole fraction reached ~86 ppt for CF_4_ and ~4.9 ppt for C_2_F_6_ in 2020 according to observations from the Advanced Global Atmospheric Gases Experiment (AGAGE) (4). Global emissions of CF_4_ and C_2_F_6_, derived using global background measurements and an inverse modeling method, have increased steadily since the 2010s (4, 12), after a period of decreasing emissions of both substances since their emission peaks in ~1980 for CF_4_ and ~2000 for C_2_F_6_ (11–13). These recent increases in emissions are occurring despite efforts from the semiconductor and aluminum industries to reduce the emissions in recent decades (7, 9, 14–16). Combined global CF_4_ and C_2_F_6_ emissions reached 138 Mt CO_2_-eq (15 Gg y^−1^ of CF_4_ and 2.2 Gg y^−1^ of C_2_F_6_) in 2020 (4, 12). A previous study suggested that the effectiveness of emission reduction measures implemented by the semiconductor and flat-panel display industries may be overestimated and attributed the global increase of CF_4_ and C_2_F_6_ in recent years to the emissions from expanding aluminum and semiconductor industries in East Asia, including China (9).

Emissions of PFCs in China from the expanding aluminum industry [especially due to low voltage anode effects (9, 16–18) and continuous emissions (16)] and perhaps from the rare earth metal industry (which uses similar processes) (9, 19), as well as from the semiconductor and flat-panel display industries, are important to understand the global CF_4_ and C_2_F_6_ budget. Several previous studies have focused on the emissions of CF_4_ and C_2_F_6_ in China. Large discrepancies exist between different bottom–up (industry data-based) emission inventories (8, 9, 20–26) and top–down (atmospheric observation-based) emission estimates (7, 9, 27–30) in China. Top–down emission estimates based on atmospheric observations can provide useful information to evaluate and improve national bottom–up inventories (17). However, existing long-term top–down estimates of CF_4_ and C_2_F_6_ emissions in China are based on measurements made outside of China (specifically in South Korea) (7, 9), which lack sensitivity to the western regions of China.

In this study, the emissions of CF_4_ and C_2_F_6_ in China (not including emissions from Hong Kong, Macao, Taiwan, and the ocean areas, throughout this study) over 2011 to 2021 were derived using long-term atmospheric observations from nine sites within China and a top–down inverse modeling technique. The measurement sites are distributed throughout China and provide good sensitivity to emissions across China. The derived spatial distributions of emissions in China are used to investigate the potential source sectors for the emissions. Finally, CF_4_ and C_2_F_6_ emission increases in China are discussed in the context of the increases in global emissions.

## Results

### Emissions of CF_4_ and C_2_F_6_ in China.

**Fig. 1.**
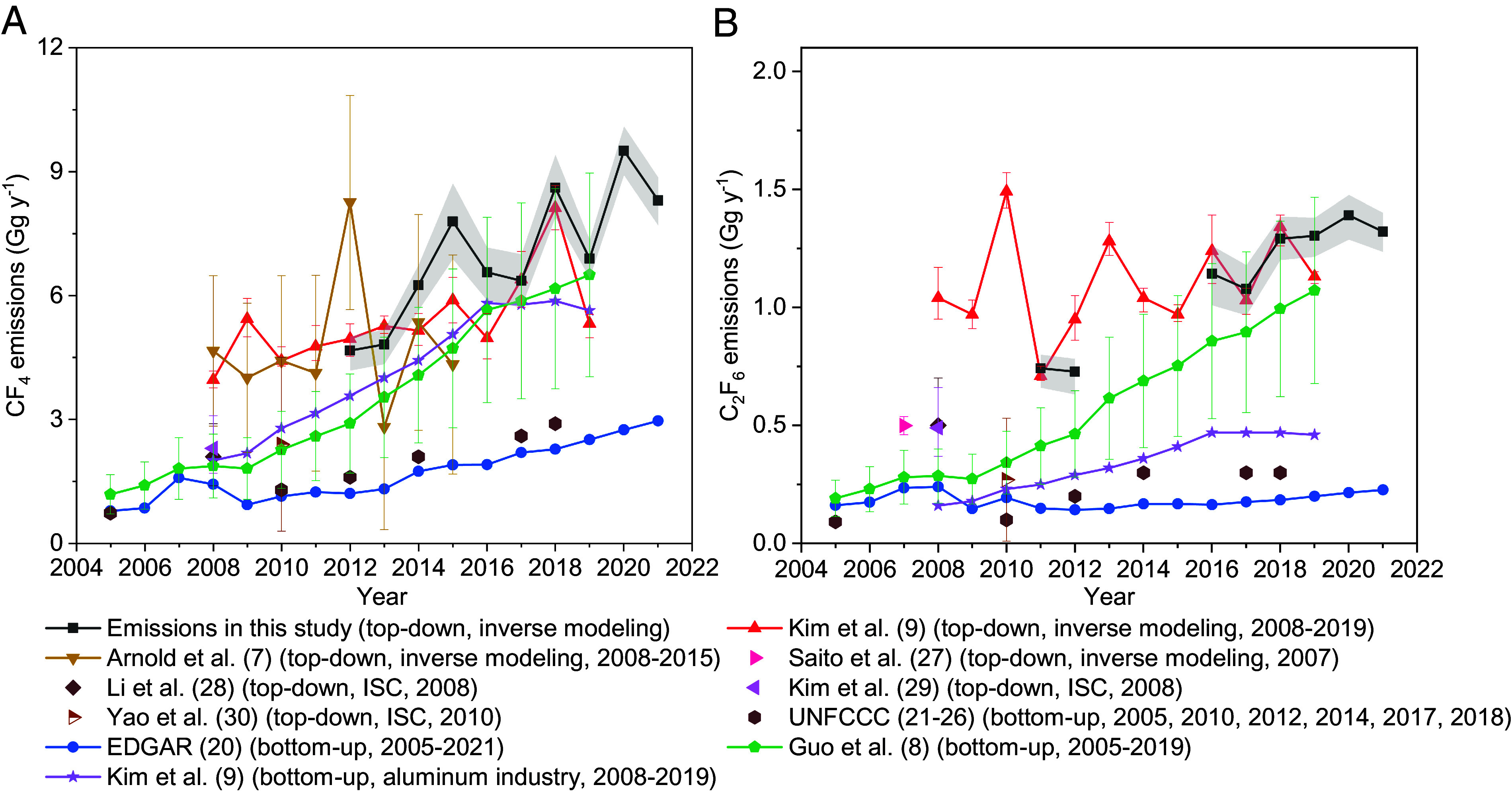
Emissions of CF_4_ and C_2_F_6_ in China. Emissions of (*A*) CF_4_ and (*B*) C_2_F_6_ derived in this study using nine sites in China (black line with its shading representing the 68% uncertainty interval) are compared to previous top–down (7, 9, 27–30) and bottom–up (8, 9, 20–26) emission estimates. Note that emissions of CF_4_ and C_2_F_6_ derived in this study are not available for some years during 2011 to 2021 for different experimental reasons (*Methods*). The years in the brackets after the legends of the previous studies are the time coverages of these studies. All available results during 2005 to 2021 were included in the plots for a complete comparison, although the previous emissions in early years do not overlap with the time coverage of this study and are not discussed. Some of the previous top–down studies were conducted using an interspecies correlation (ISC) method. The CF_4_ and C_2_F_6_ emissions derived in this study are tabulated in *SI Appendix,* Table S1 and Dataset S1.

Emissions of both CF_4_ and C_2_F_6_ in China derived in this study show substantial increases over 2011 to 2021 (Fig. 1). Emissions of CF_4_ increased from 4.7 (4.2-5.0, 68% uncertainty interval, the same hereafter) Gg y^−1^ in 2012 to 8.3 (7.7-8.9) Gg y^−1^ in 2021, a relative increase of ~78%. Emissions of C_2_F_6_ increased from 0.74 (0.66-0.80) Gg y^−1^ in 2011 to 1.32 (1.24-1.40) Gg y^−1^ in 2021, a relative increase of ~78%. These derived (posterior) emissions of CF_4_ and C_2_F_6_ are relatively insensitive to the a priori emissions magnitudes and prior probability distributions (*SI Appendix,* Fig. S1) and also to the choices of measurement datasets (*SI Appendix,* Fig. S2) that were used in the inversion, both in terms of the a posteriori emission magnitudes in each specific year and the general trend.

Several previous studies (7–9, 20–30) have quantified emissions of CF_4_ and C_2_F_6_ in China, and substantial increases in CF_4_ and C_2_F_6_ emissions have been identified in some of these studies [e.g., Guo et al. (8) and Kim et al. (9)] (Fig. 1). However, large discrepancies exist between previous bottom–up and top–down emissions. The top–down CF_4_ and C_2_F_6_ emissions derived in this study agree reasonably with previously published top–down emissions, given some of the large uncertainties (7, 9). The relative differences between previous top–down studies (7, 9) and the mean estimates of this study (~24% for CF_4_ emissions and ~11% for C_2_F_6_ emissions, on average over the overlapping years) may result from the different atmospheric measurements used and perhaps also from the different inverse modeling approaches taken. These top–down emission time series [from this study and previous studies (7, 9)] commonly exhibit significant interannual variations, which may be genuine or an artifact due to the model-measurement errors. The top–down CF_4_ and C_2_F_6_ emissions derived in this study are relatively close to a recently published bottom–up inventory of Guo et al. (8), especially in recent years when they agree within uncertainties, although the best estimates of Guo et al. (8) are still lower (e.g., ~6% relatively lower for CF_4_ emissions and ~18% relatively lower for C_2_F_6_ emissions in 2019) than the mean values of our top–down emissions. Our top–down emissions also agree relatively well with (but are still, on average, ~30% larger than) a bottom–up inventory for CF_4_ emissions from the aluminum industry (9). The top–down CF_4_ and C_2_F_6_ emissions derived here are substantially larger than the bottom–up Emissions Database for Global Atmospheric Research (EDGAR) v8.0 inventory (20) (with the EDGAR inventory accounting for a range of only ~24-36% of the top–down CF_4_ emissions derived in this study and ~14-20% of the top–down C_2_F_6_ emissions in this study during 2011 to 2021) and the officially reported bottom–up emissions from China’s national communications or biennial update to the UNFCCC (23–26) (with officially reported emissions accounting for a range of only ~34-41% of top–down CF_4_ emissions in this study and ~23-28% of top–down C_2_F_6_ emissions in this study over the overlapping years). The substantially lower bottom–up inventories compared to the top–down emissions indicate that these current bottom–up inventories are likely underestimated, in terms of the magnitudes of production of the relevant industries and/or the emission factors.

### Spatial Distributions of Emissions of CF_4_ and C_2_F_6_ in China.

**Fig. 2.**
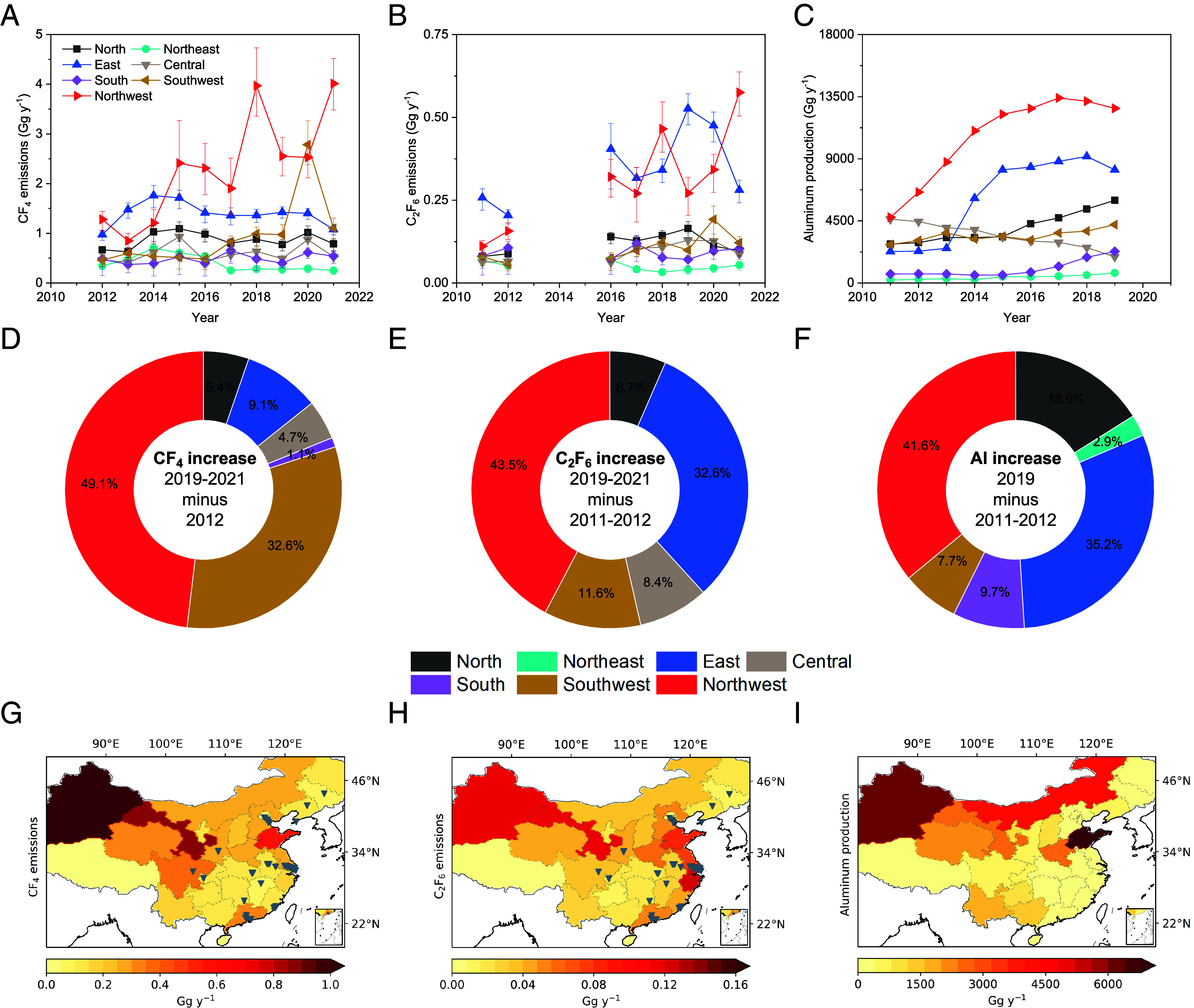
Emissions of CF_4_ and C_2_F_6_ and aluminum production in different subregions of China. The definitions of each subregion can be found in *SI Appendix,* Fig. S3. Panel (*A*) shows CF_4_ emissions, (*B*) C_2_F_6_ emissions, and (*C*) aluminum production in each subregion; panel (*D*) shows the increase in emissions of CF_4_ between 2012 and the 2019-2021 average, (*E*) the increase in emissions of C_2_F_6_ between the 2011-2012 and 2019-2021 averages, and (*F*) the aluminum production increase between the 2011-2012 average and 2019, from each subregion; panel (*G*) shows the spatial distribution of CF_4_ emissions, (*H*) C_2_F_6_ emissions and (*I*) aluminum production in each province (not including data for Hong Kong, Macao, Taiwan, and ocean regions). The uncertainties for CF_4_ and C_2_F_6_ emissions in plots (*A* and *B*) are the 68% uncertainty intervals. The values of subregional CF_4_ and C_2_F_6_ emissions can be found in Dataset S1. The sum of the percentages in plots (*D*–*F*) may be larger than 100%, because emissions/production in some of the regions may have decreased. The aluminum production data were obtained from the Yearbooks (31), with the subregional aluminum production in each year tabulated in *SI Appendix,* Table S2. Note that aluminum production data are only available during 2011 to 2019. The values shown in plots (*G*–*I*) represent the total annual quantities in each province averaged over 2016 to 2019, a period when emissions of CF_4_ and C_2_F_6_ and aluminum production data are all available. The gray triangles in plots (*G* and *H*) are the locations of semiconductor factories, obtained from Wikipedia (https://en.wikipedia.org/wiki/List_of_semiconductor_fabrication_plants, last access: 16 April 2023). The spatial distributions for all years can be found in *SI Appendix,* Figs. S4–S8.

We divide the emissions of CF_4_ and C_2_F_6_ in China into seven subregions, as shown in Fig. 2. The northwest of China contributes the most to the national total CF_4_ emissions in most years, a region for which previous top–down studies (7, 9) had low sensitivities. For emissions of C_2_F_6_, the northwest and east of China contribute most to the national total emissions among all the regions over the study period. We note large interannual variations in some subregional emissions, possibly due to the weaker constraint from the limited number of observations in these subregions. Thus, we used multiyear averaging over different periods to investigate the long-term trend of emissions in each subregion. Emissions from the northwest of China show substantial increases of 1.8 (1.4-2.1) Gg y^−1^ for CF_4_ and 0.26 (0.22-0.30) Gg y^−1^ for C_2_F_6_ between 2011-2012 and 2019-2021. The northwest of China contributed most to the national total emissions increase during this period (~49.1% of the national emissions rise for CF_4_ and ~43.5% for C_2_F_6_) (Fig. 2 *D* and *E*). The northwest of China is generally less populated and less economically developed than other regions of China. However, this resource-intensive region has the largest aluminum production and contributes most to the aluminum production increase in China (Fig. 2 *C* and *F*). Both CF_4_ and C_2_F_6_ are formed as a by-product during anode effects in the aluminum industry and during continuous operations (16–18), likely explaining the substantial emissions in the northwest of China. The east of China also contributes significantly to national total C_2_F_6_ emissions and their increases in China (Fig. 2 *B* and *E*). The east of China is a highly populated and economically developed region with substantial aluminum production (Fig. 2 *C* and *I*), significant presence of semiconductor industry (gray triangles in Fig. 2 *G* and *H* and *SI Appendix,* Fig. S9*A*), and perhaps substantial flat-panel display industry (*SI Appendix,* Fig. S9*B*).

From the plots of emission spatial distributions (Fig. 2 *G*–*I*), emissions of CF_4_ are located largely in the northwest regions of China, especially in recent years (*SI Appendix,* Figs. S4 and S5), and the distributions of CF_4_ emissions are highly consistent with distributions of aluminum production. The spatial distributions of C_2_F_6_ emissions (Fig. 2*H*) are similar to those of aluminum production (Fig. 2*I*), while some of the largest emissions of C_2_F_6_ are colocated with semiconductor (Fig. 2 *G* and *H* and *SI Appendix,* Fig. S9*A*) and flat-panel display (*SI Appendix,* Fig. S9*B*) industries (e.g., around the Yangtze River Delta region in the east of China). The spatial distribution features of CF_4_ and C_2_F_6_ emissions imply that aluminum production is likely the dominant source of CF_4_ emissions in China, including its significant contribution to emissions in the western regions of China, while aluminum production and the semiconductor (and flat-panel display) industry are both major sources of C_2_F_6_ emissions, probably from different regions. This can also be validated to some extent by the comparison between emissions in this study with a previous bottom–up inventory from the aluminum industry [bottom–up emissions from Kim et al. (9) in Fig. 1]. The top–down emissions of CF_4_ in this study are relatively close to the bottom–up CF_4_ emissions from the aluminum industry (9), while the top–down emissions of C_2_F_6_ in this study are much larger than the estimated bottom–up C_2_F_6_ emissions from aluminum industry (9) (Fig. 1), which is expected given the relative industry contributions to CF_4_ and C_2_F_6_ emissions in China identified above. In this study, we do not explicitly discuss emissions from the rare earth metal industries due to the lack of industry data. However, the source processes of CF_4_ and C_2_F_6_ from the rare earth metal industry are similar to those of the aluminum industry, and the contribution from the rare earth industries to CF_4_ and C_2_F_6_ emissions may be relatively small (9, 19), though more studies are needed.

**Fig. 3.**
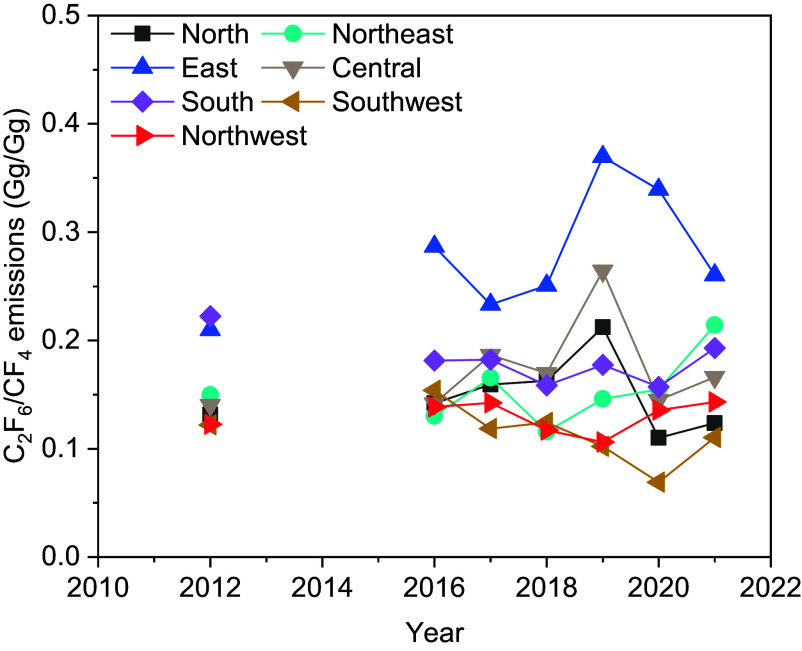
Emission ratios of C_2_F_6_/CF_4_ in each subregion of China. Due to the lack of CF_4_ emissions in 2011 and C_2_F_6_ emissions in 2013 to 2015, only ratios in 2012 and over 2016 to 2021 are calculated.

The emission ratio of C_2_F_6_/CF_4_ can provide insights into the origin of emissions in a specific region. This emission ratio is generally < 0.1 from the aluminum industry (and the rare earth metal industry) and > 0.4 from the semiconductor industry (including the flat-panel display industry) (9, 10, 17). The emission ratio in each subregion in each year is shown in Fig. 3. Emission ratios of C_2_F_6_/CF_4_ in the northwest and southwest regions are among the lowest values and close to 0.1, suggesting that the CF_4_ and C_2_F_6_ emitted in these western regions originate mostly from the aluminum industry. The average emission ratio of C_2_F_6_/CF_4_ in the east of China over the study period is 0.28, the highest among all the regions, and is between the reported emission ratios from the aluminum industry and semiconductor (and flat-panel display) industry, suggesting comparable contributions of the two industries to CF_4_ and C_2_F_6_ emissions in the east of China.

The substantial emissions of CF_4_ and C_2_F_6_ in the western regions likely result from the expanding aluminum industry in this resource-intensive area. Monitoring CF_4_ and C_2_F_6_ emissions in the western regions is necessary to evaluate the effectiveness of controls to reduce emissions of greenhouse gases and the achievement of carbon neutrality goals. Two previous long-term top–down emissions estimates of CF_4_ and C_2_F_6_ were derived by measurements made outside of China (7, 9), specifically from the Gosan AGAGE site located in South Korea, which has lower sensitivity to emissions in regions beyond eastern China, e.g., the northwestern regions of China (*SI Appendix,* Fig. S10). Although the derived emissions from these previous top–down studies are relatively close to our top–down emissions (Fig. 1, ~24% relative difference on average over the overlapping years compared to this study for CF_4_ emissions and ~11% relative difference for C_2_F_6_ emissions), the two sites across the northwest of China used in this study (WLG and AKD, see *Methods*) likely provided additional information to constrain the spatial distributions of emissions. When comparing previous top–down emissions to those in this study, lower emissions were more commonly observed for CF_4_ than C_2_F_6_ (Fig. 1), which could be due to the substantial CF_4_ emissions in the western regions emitted from the aluminum industries that were not well constrained by the measurements used previously. Without using the two sites in the northwest of China in this study (*SI Appendix,* Fig. S11), the derived emissions in China change by an average of ~12% over the period for CF_4_, and ~9% for C_2_F_6_, compared to emissions derived using all sites. Although excluding the sites in the northwest of China does not make a large difference to our conclusions drawn on the overall emission magnitudes and general trends in China, some of the spatial distribution information in the western regions would be missing if no sites in the northwest were used (*SI Appendix,* Fig. S11 *C* and *D*).

### Emissions in China and Globally.

**Fig. 4.**
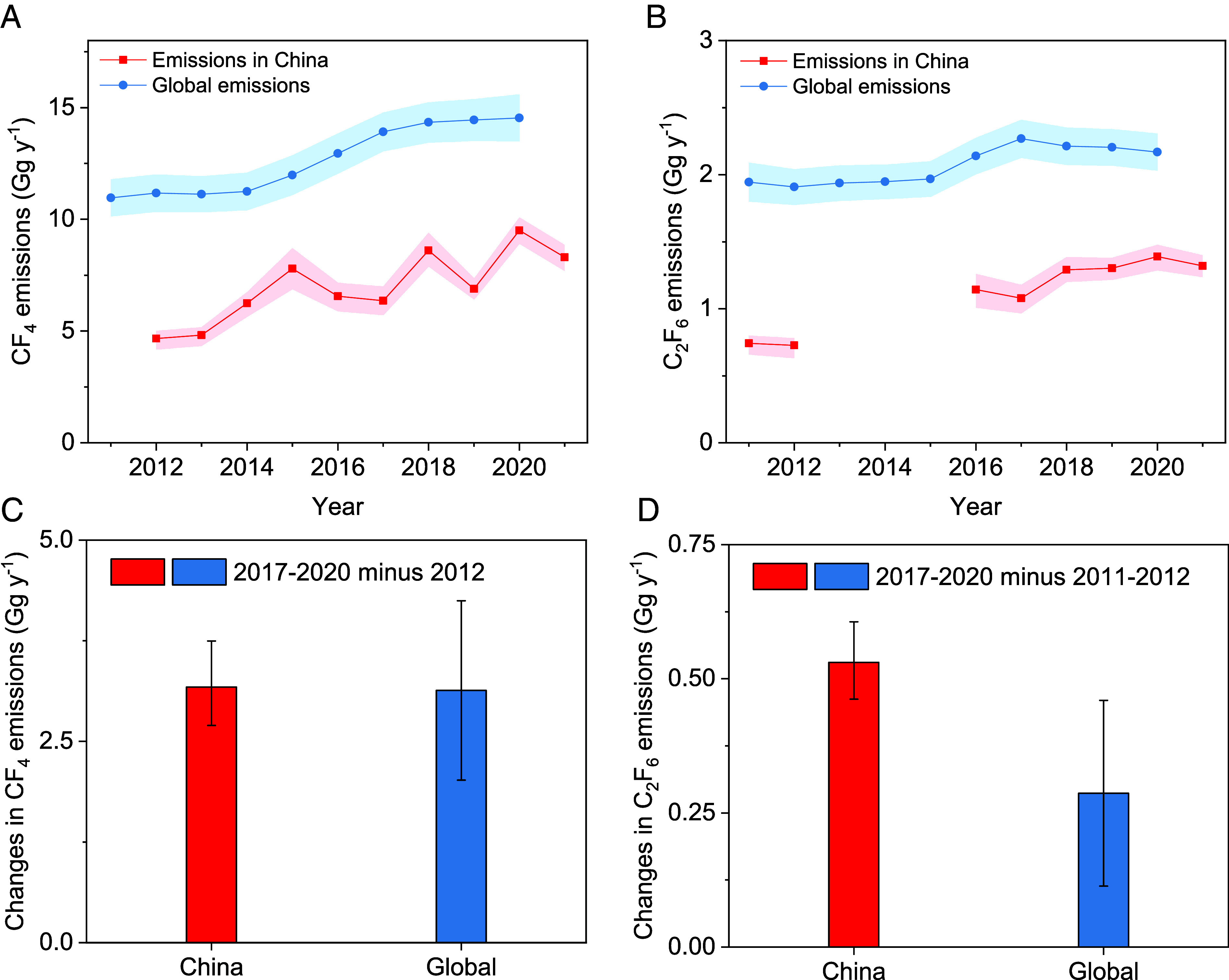
Emissions of CF_4_ and C_2_F_6_ in China compared to global emissions. Panel (*A*) shows a comparison between the emissions of CF_4_ and (*B*) C_2_F_6_ in China derived in this study and the global emissions obtained from Laube and Tegtmeier (4) from AGAGE data. Multiyear averages were used to calculate the increase in emissions in China and globally. Panel (*C*) shows the changes in emissions between 2012 and the average of 2017-2020 for CF_4_ and (*D*) between the average of 2011-2012 and the average of 2017-2020 for C_2_F_6_.

Emissions of CF_4_ and C_2_F_6_ in China derived in this study are compared to global CF_4_ and C_2_F_6_ emissions (4) (Fig. 4 and *SI Appendix,* Table S1). The CF_4_ and C_2_F_6_ emissions in China and globally increased substantially between 2011/12 and 2020. Emissions in China represented a substantial fraction of global emissions during 2011 to 2020 and explained a major fraction of the global trend. Emissions of CF_4_ from China were responsible for 42 (36-46)% of the global total CF_4_ emissions in 2012, reaching 66 (59-71)% in 2020. Emissions of C_2_F_6_ in China were responsible for 38 (33-42)% of the global total C_2_F_6_ emissions in 2011, reaching 64 (58-70)% in 2020. The increase in CF_4_ emissions in China is nearly equal to the global CF_4_ emission increase between 2012 and 2017-2020 [Fig. 4*C*, an increase of 3.2 (2.7-3.7) Gg y^−1^ was derived for CF_4_ emissions in China between the two periods, compared to 3.1 (2.0-4.2) Gg y^−1^ globally]. The increase in C_2_F_6_ emissions in China is larger than the global C_2_F_6_ emission increase between 2011-2012 and 2017-2020 [Fig. 4*D*, an increase of 0.53 (0.46-0.61) Gg y^−1^ from China between these periods, compared to 0.29 (0.11-0.46) Gg y^−1^ globally], suggesting that C_2_F_6_ emissions may have decreased elsewhere in the world over the same period. It is worth noting that the increases in C_2_F_6_ emissions from China and globally are both highly uncertain.

The global C_2_F_6_/CF_4_ emission ratio ranges between about 0.15 to 0.18, depending on the year, which closely mirrors the C_2_F_6_/CF_4_ emission ratio of around 0.15 to 0.19 observed in China (*SI Appendix,* Fig. S12). This suggests that the relative size of emissions from different industrial sources of CF_4_ and C_2_F_6_ is similar in China and globally, with both the aluminum and the semiconductor/flat-panel display industries being the major contributors. The global C_2_F_6_/CF_4_ emission ratios exhibit a decreasing trend over 2011 to 2020, indicating a global shift toward a greater dominance of aluminum industry in CF_4_ and C_2_F_6_ emissions compared to the semiconductor (and flat-panel display) industries and/or the gradual replacement of C_2_F_6_ by alternatives such as NF_3_ in certain cleaning processes in the semiconductor industries (9, 32). The ratios of C_2_F_6_/CF_4_ emissions in China show a large scatter with no discernable trend, perhaps in part due to the lack of data for several years (*SI Appendix,* Fig. S12). Primary aluminum production in China exhibits nearly identical growth patterns to the global total aluminum production over the last decade and contributes more than 90% to the global total increase in aluminum production (*SI Appendix,* Fig. S13). The dominance of China in the increase in global aluminum production is likely, in part, driving the global emissions growth of CF_4_ and C_2_F_6_. The slowdown of the C_2_F_6_ emission increase since 2018 in China and globally could be attributed to the emission reductions from the semiconductor/flat-panel display industries in recent years, where NF_3_ is replacing C_2_F_6_ in cleaning applications (9, 32), while the lack of abatement equipment for destroying unused gases in these industries during the early years in China could be a potential explanation for the greater C_2_F_6_ emission increase in China (best estimate) compared to the global total increase in C_2_F_6_ emissions. Unfortunately, no further information from the semiconductor and flat-panel display industries is available to validate this hypothesis.

## Discussion

Top–down emission estimates based on atmospheric observations can help to evaluate and improve national bottom–up inventory reporting and provide insights into spatial distributions and emissions source sectors. In this study, using long-term atmospheric observations of CF_4_ and C_2_F_6_ from a Chinese measurement network and an inverse modeling technique, we inferred rapid increases in CF_4_ and C_2_F_6_ emissions from China over the last decade. The emissions from China contributed to a range of ~42-66% of global total CF_4_ emissions and ~38-64% of global C_2_F_6_ emissions, depending on the year during the study period. The absolute increases in CF_4_ and C_2_F_6_ emissions in China are of similar magnitude (for CF_4_) or larger than (for C_2_F_6_) the absolute increases in global emissions over the same period, suggesting that changes in emissions in China may be the dominant driver behind changes in global CF_4_ and C_2_F_6_ emissions. We are also able to explore spatial distributions of the emissions sources. Substantial emissions of CF_4_ and C_2_F_6_ were determined to come from the less populated and less economically developed western regions of China. Emissions from these western regions of China are likely dominated by emissions from the aluminum industry, while the semiconductor and flat-panel display industries are likely to be considerable sources of emissions in the more economically developed regions, especially for C_2_F_6_.

The combined emissions of CF_4_ and C_2_F_6_ in China reached 78 Mt CO_2_-eq in 2021, which is equivalent to ~0.6-0.7% of the national total CO_2_ emissions (20, 33), ~4% of the national total CH_4_ emissions (20) (using GWP_100_ for CH_4_ of 27.9), and around one-fifth of the national total N_2_O emissions (20) (using GWP_100_ for N_2_O of 273) in 2021. The ongoing CF_4_ and C_2_F_6_ emissions in China, especially under the rapid expansion of China’s aluminum and semiconductor industries, could offset progress toward China’s carbon neutrality goal and global climate mitigation. There is a significant potential for reductions of CF_4_ and C_2_F_6_ emissions in China through technological innovation in the aluminum industry under different scenarios (34). The possibility of incorporating the aluminum industry into the carbon market in China (e.g., ref. 35) could stimulate the emission reductions of CF_4_ and C_2_F_6_. It is important to continue to monitor emissions of CF_4_ and C_2_F_6_ in China, including in the resource-intensive western regions, to validate national inventory reporting and evaluate the effectiveness of emission mitigation controls.

## Methods

To estimate CF_4_ and C_2_F_6_ emissions in China, we utilized the Numerical Atmospheric-dispersion Modelling Environment (NAME)-hierarchical Bayesian inference with Markov chain Monte-Carlo method (HBMCMC) regional inverse modeling framework that is described in detail in several previous papers (36–40). The framework has been used to estimate regional emissions from both in situ and flask measurements at multiple sites in a Chinese network (36, 39, 40). The approach consists of four parts: atmospheric observations, sensitivities of the observations to regional emissions, prior estimates of the emissions, and a Bayesian inference algorithm to solve for the emissions.

### Atmospheric Observations.

Atmospheric mole fractions of CF_4_ and C_2_F_6_ were measured at nine sites in China, as part of the China Meteorological Administration (CMA) network. The sites are located in different regions of China, namely Akedala (AKD, 47.10° N 87.97° E) and Mt. Waliguan (WLG, 36.29° N 100.90° E) in the northwest of China, Lin’an (LAN, 30.30° N 119.73° E) in the east of China, Longfengshan (LFS, 44.73° N 127.60° E) in the northeast of China, Jiangjin (JGJ, 29.15° N 106.15° E) and Shangri-La (XGL, 28.01° N 99.44° E) in the southwest of China, Jinsha (JSA, 29.64° N 114.21° E) in central China, Shangdianzi (SDZ, 40.65° N 117.12° E) in the north of China, and Xinfeng (XFG, 24.08° N 114.17° E) in the south of China. Two modes of sampling were conducted at the sites: namely flask samplings and high-frequency in situ samplings (*SI Appendix,* Table S3). Weekly flask sampling was conducted at SDZ, WLG, LFS, XGL, AKD, XFG, and JSA, and daily flask sampling was conducted at JGJ. A mix of weekly and daily flask samples were collected at LAN (weekly before 2018 and daily after 2019). All the flask samples were analyzed at the CMA Beijing Laboratory. In addition to the above flask sampling, ~two-hourly in situ measurements were conducted at the SDZ station during 2011 to 2012 and 2016 to 2021. These measurements made at the above sites can in total provide good sensitivity to CF_4_ and C_2_F_6_ emissions across China (*SI Appendix,* Figs. S14 and S15).

AGAGE Medusa gas chromatographic system with mass spectrometric detector (41, 42) at the CMA Beijing Laboratory was used to analyze the mole fractions of CF_4_ and C_2_F_6_ from the flask samples and a second Medusa system was used for the in situ measurements at SDZ. The mole fractions are reported on the AGAGE SIO-14 calibration scale (43). The analyses of the samples were bracketed by analyses of the working standard gases to calibrate the measurements. The precision of the measurements is estimated to be 0.5% and 1% for in situ measurements and flasks for CF_4_, respectively, and 0.9% and 1.5% for C_2_F_6_. Further details regarding the sampling and measurements are available in previous studies (30, 44, 45). The high-frequency in situ measurements were averaged every 24 h in the inversion framework to reduce the influence of correlated model uncertainties between the successive samples. The observations for CF_4_ and C_2_F_6_ can be found in Datasets S2 and S3. The mole fractions that were input to the inversion framework after resampling are shown in *SI Appendix,* Figs. S16 and S17.

### Sensitivities of the Observations to Emissions.

To derive emissions based on the atmospheric observations, the quantitative relationships (sensitivities) between the observations and emissions are needed. The sensitivities of the atmospheric observations to emissions in each grid were estimated using a Lagrangian particle dispersion model, the UK Met Office NAME (46), which was run backward in time for 30 d to calculate the interaction of the air with the surface, and thus the sensitivity of the observations to surface emissions. Particles located within the lowest 40 m of the atmosphere above ground level were regarded as interacting with the surface emissions. The detailed configuration for the NAME runs can be found in previous studies (36–40). The output grid has a spatial resolution of ~0.234° in latitude and ~0.352° in longitude, within a computational domain between 5° S, 74° N, and 55° E, 192° E. These grids are aggregated into 150 regions by applying a quadtree algorithm (47) to the a priori contribution from each grid to the mole fractions. The 150 regions were used as the basic calculation units in the inverse modeling (basis functions).

The regional emissions are estimated based on the enhancements of the mole fractions over the background values (i.e., pollution events), so an estimate of background values is also needed. In the NAME-HBMCMC framework, the background is defined as the influence of all emissions outside of the computational domain as well as the well-mixed global background mole fractions. In the NAME backward run, the locations of particles leaving the computational domain were integrated to calculate the sensitivities of the observations to the background values.

### Bayesian Inference Algorithm and Prior Emissions.

A priori information is needed to estimate the emission values in the Bayesian inference algorithm (48, 49). The a priori magnitudes for emissions were 5 Gg y^−1^ over China for CF_4_ and 0.8 Gg y^−1^ for C_2_F_6_ for all years, which are similar to the magnitudes of emissions in previous studies (7–9). The emissions were distributed in space using the intensity of lights at night, taken from the NOAA Defense Meteorological Satellite Program-Operational Line-Scan System (https://ngdc.noaa.gov/eog/data/web_data/v4composites/, last access: 1 March 2021), used here as a proxy for anthropogenic activity. The a priori emissions in each basis function were scaled during the inversion to optimally fit the observations, where the scaling factor was assumed to follow a lognormal distribution, with shape parameters mu of 0.2 and sigma of 0.8. This prior probability distribution ensures that the derived emissions are only positive.

The a priori magnitudes for background values were estimated by multiplying the NAME sensitivities to background values calculated above, by a background concentration field at the domain edges, which were taken from global AGAGE 12-box model inversions (50, 51). These estimates of background concentration field are based on the assimilation of monthly mean measurements at long-running background AGAGE sites. The calculated a priori background values were scaled during the inversion and are referred to as the boundary conditions in the framework. The prior probability distribution for the scaling factors of the boundary conditions was assumed to follow a lognormal distribution, with shape parameters mu of 1 and sigma of 1. A Markov chain Monte-Carlo method was used to solve the Bayesian inference equation, as explained in previous studies (36, 37).

The number of available observations in each year of 2013 to 2015 for C_2_F_6_ (as shown in *SI Appendix,* Fig. S17), is smaller than the number of basis functions (150), and there is only in situ data from SDZ available for CF_4_ in 2011 (as shown in *SI Appendix,* Table S3). Due to the scarcity of data in these specific years, the emissions cannot be effectively constrained. Thus, only the emissions of CF_4_ over 2012 to 2021, and the emissions of C_2_F_6_ in 2011 to 2012 and 2016 to 2021 are discussed in this study, as shown in Fig. 1.

## Supplementary Material

Appendix 01 (PDF)

Dataset S01 (XLSX)

Dataset S02 (XLSX)

Dataset S03 (XLSX)

## Data Availability

The observations from nine Chinese sites used to derived CF_4_ and C_2_F_6_ emissions in China can be found in Datasets S2 and S3. Prior discussions with B.Y. about interest of using these data in future publications or presentations are required. The code for the regional inverse modeling framework “NAME-HBMCMC” is available from An (2024) (52) https://doi.org/10.5281/zenodo.10929382.
