# Supplementary material for: Substantial increase in perfluorocarbons CF4 (PFC-14) and C2F6 (PFC-116) emissions in China
Source: Proc Natl Acad Sci U S A. 2024 Jul 15;121(30):e2400168121. doi: 10.1073/pnas.2400168121 (PMC11287116; doi:10.1073/pnas.2400168121)
Supplement: Supplementary file 1 — Appendix 01 (PDF) [file pnas.2400168121.sapp.pdf]

PNAS

Supporting Information for
Substantial increase in perfluorocarbons CF_4 (PFC-14) and C_2F_6 (PFC-116) emissions in China

Minde An, Ronald G. Prinn, Luke M. Western, Bo Yao, Xingchen Zhao, Jooil Kim, Jens Mühle, Wenxue Chi, Christina M. Harth, Jianxin Hu, Anita L. Ganesan, Matthew Rigby

Corresponding authors: Minde An; Bo Yao
Email: mindean@mit.edu; yaobo@fudan.edu.cn

This PDF file includes:

Figures S1 to S17
Tables S1 to S3
Legends for Datasets S1 to S3
SI References

Other supporting materials for this manuscript include the following:

Datasets S1 to S3

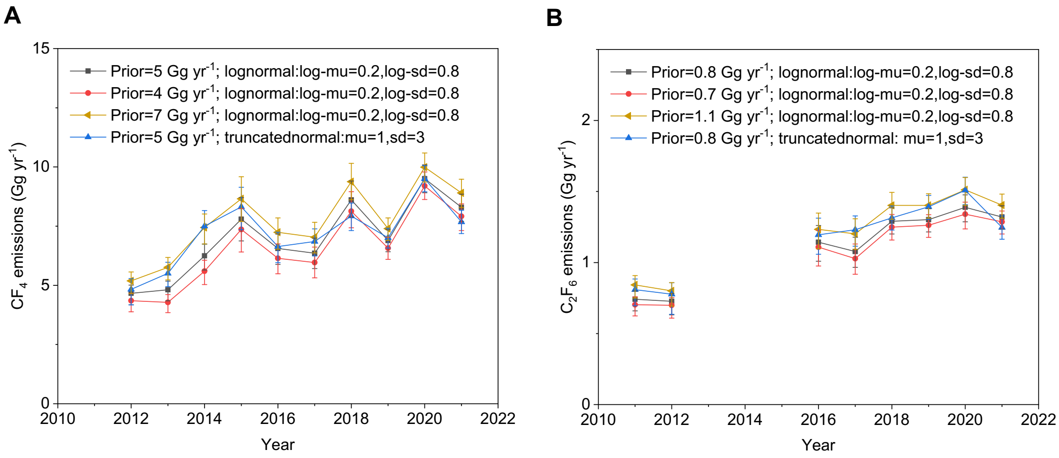

Fig. S1. Inversions of CF_4 and C_2F_6 using different a priori emission magnitudes and prior probability distributions. (A) Sensitivity tests for CF_4. (B) Sensitivity tests for C_2F_6. The lognormal is the probability distribution used in the main text, with shape parameters mu=0.2 and sigma=0.8. The term "truncatednormal" refers to the inversion using a truncated-normal prior probability distribution with mean of 1, standard deviation of 3 (representing 300% uncertainty) and a lower bound at 0.

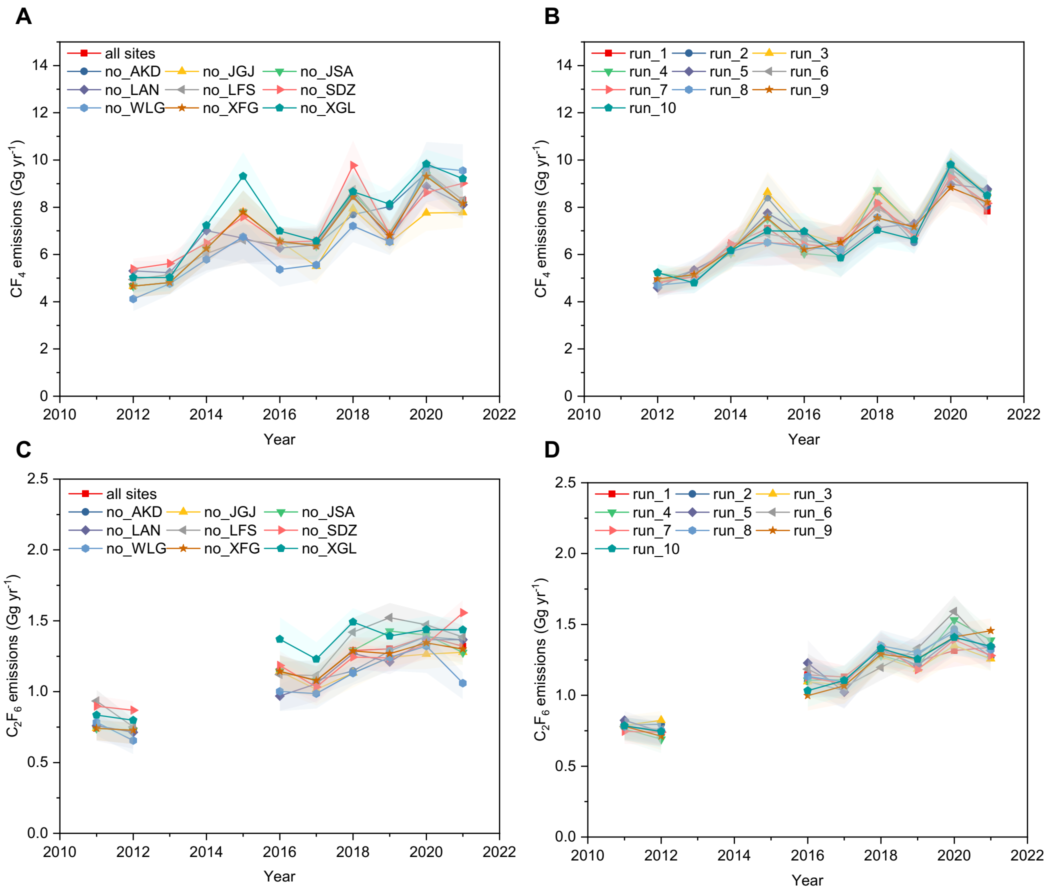

Fig. S2. Inversions of CF_4 and C_2F_6 using different choices of measurement datasets. Plots (A) and (C) are inversions conducted using different combinations of sites. In each inversion run in plot (A) and (C), measurements from one of the sites were removed in the inversion (removed site is indicated in the legend). Plots (B) and (D) are inversions using a random subset of all measurements. In each run, 80% of the total available measurements in each year were randomly subsampled and were used in the inversion, and the run was repeated 10 times. Plots (A) and (B) are inversions for CF_4. Plots (C) and (D) are for C_2F_6. The shadings represent the 68% uncertainty intervals.

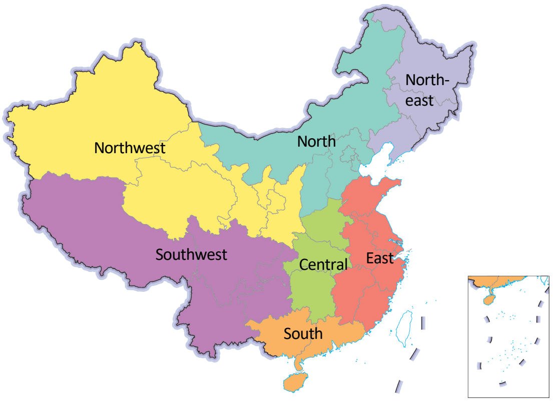

Fig. S3. Definition of subregions in China. Note that Hong Kong, Macao, Taiwan and the ocean areas are not included throughout this study. The emissions in China are divided into seven subregions: North (including Inner Mongolia, Beijing, Tianjin, Hebei and Shanxi provinces), Northwest (including Xinjiang, Gansu, Qinghai, Ningxia and Shaanxi provinces), Northeast (including Liaoning, Jilin and Heilongjiang provinces), Central (including Henan, Hunan and Hubei provinces), East (including Shandong, Jiangsu, Anhui, Shanghai, Zhejiang, Jiangxi and Fujian provinces), South (including Guangdong, Guangxi and Hainan provinces) and Southwest (including Yunnan, Guizhou, Sichuan, Chongqing and Xizang provinces).

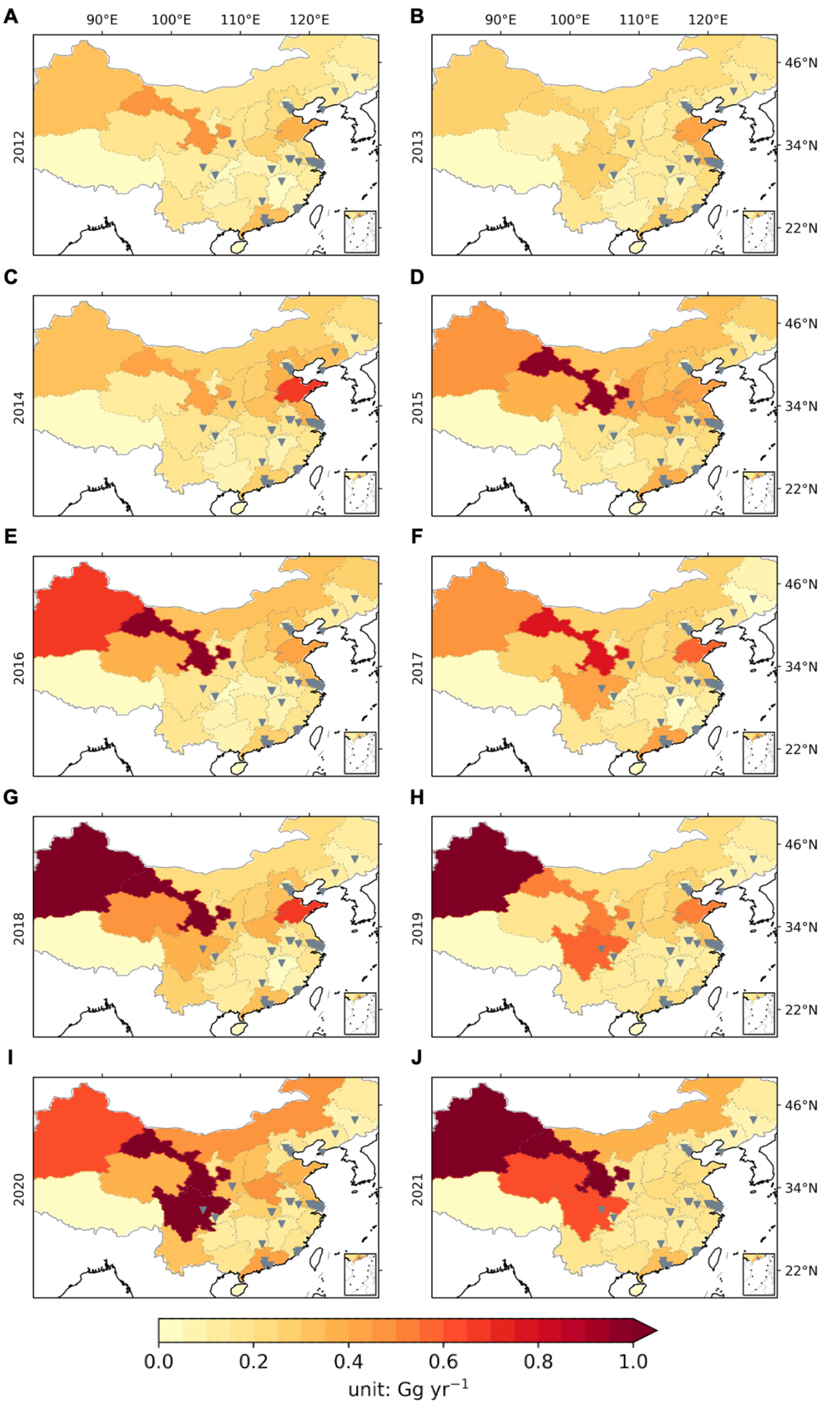

Fig. S4. Spatial distributions of CF_4 emissions in China by province. The grey triangles are the semi-conductor factories in China, the locations of which were obtained from Wikipedia (https://en.wikipedia.org/wiki/List_of_semiconductor_fabrication_plants, last access: 16 April, 2023). The values shown in the plots are total CF_4 emissions in the provinces.

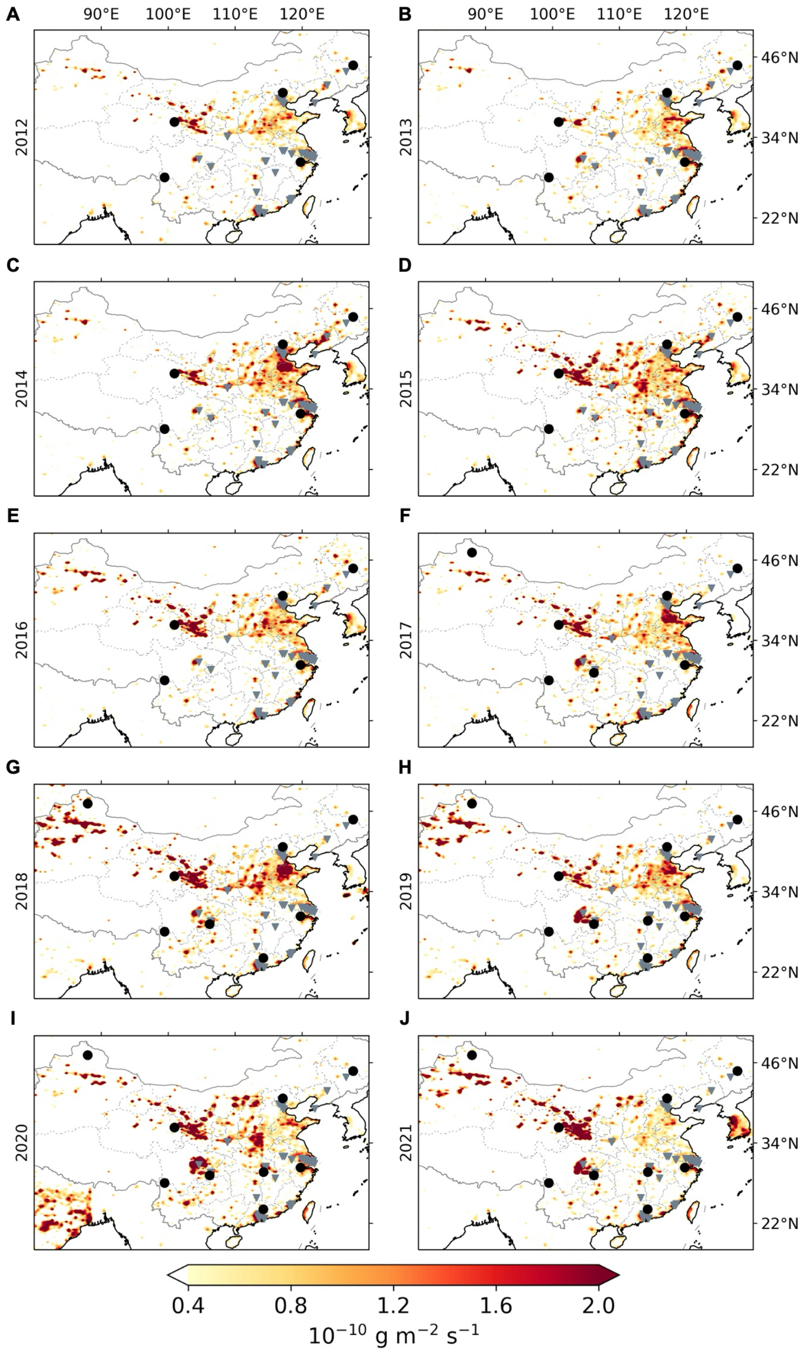

Fig. S5. Spatial distributions of CF_4 emissions in Eastern Asia with focus on China. As in Fig. S4, but illustrating the actual spatial distributions of the a posteriori emissions from the inversion (not aggregated by province). The black dots are the active measurement sites in the year.

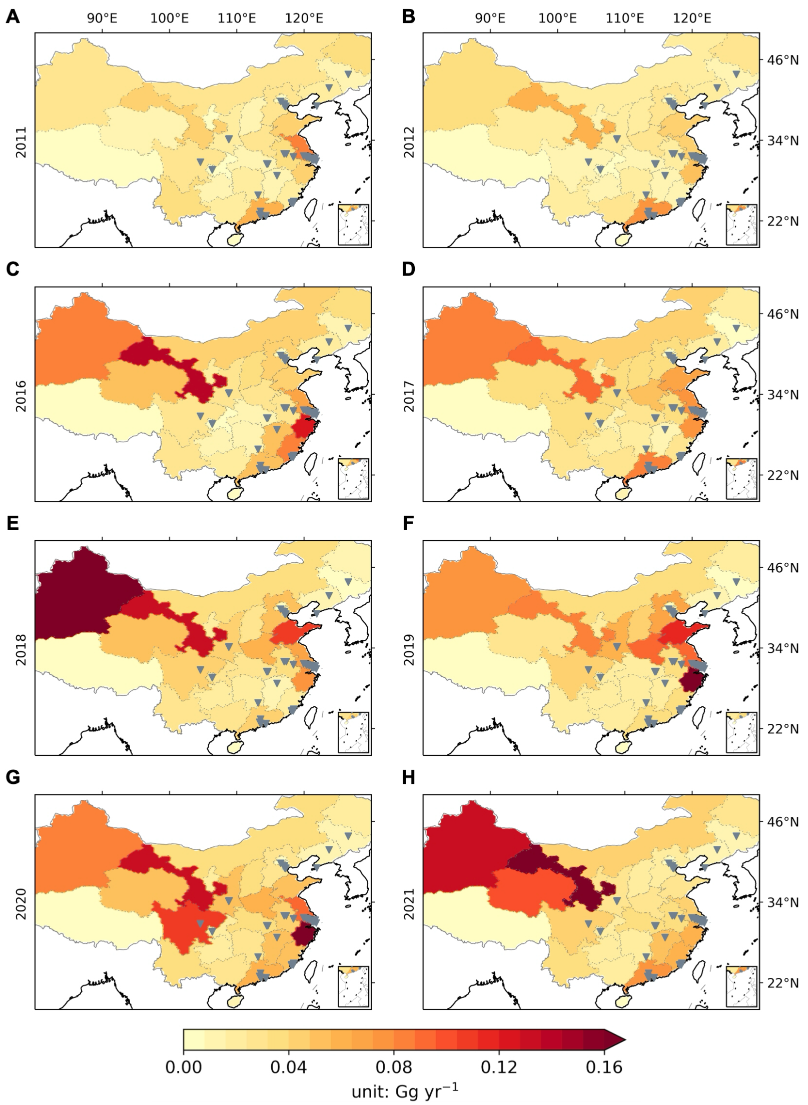

Fig. S6. Spatial distributions of C_2F_6 emissions in China by province. As Fig. S4 but for C_2F_6.

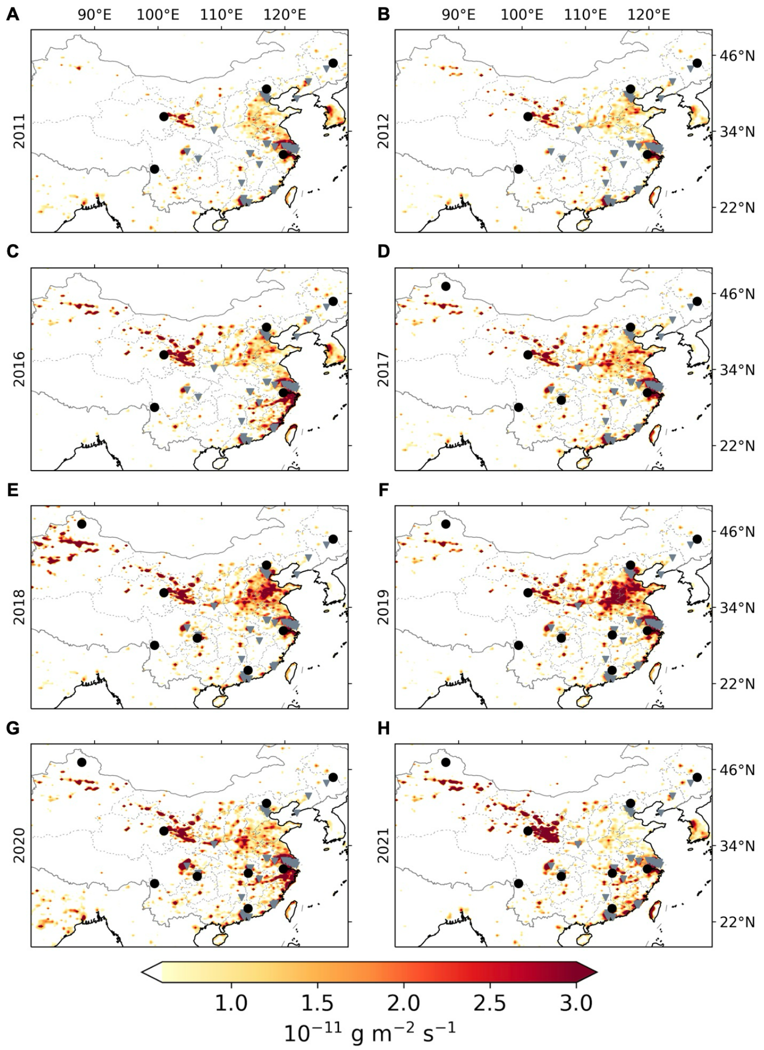

Fig. S7. Spatial distributions of C_2F_6 emissions in Eastern Asia with focus on China. As Fig. S5, but for C_2F_6.

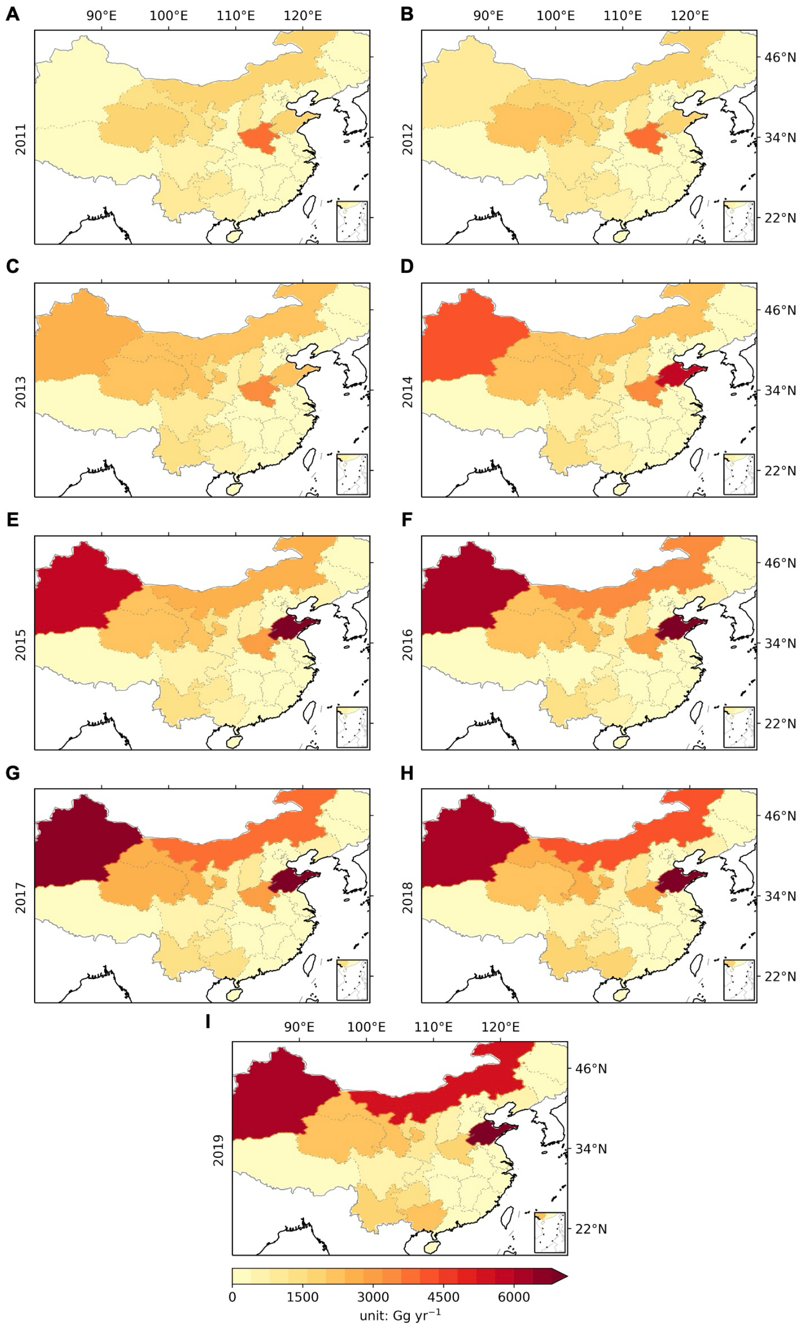

Fig. S8. Spatial distributions of aluminum production in China by province. The values shown in the plots are total aluminum production in the provinces. The aluminum production in each province over 2011-2019 was obtained from the Yearbook of Nonferrous Metals Industry of China (1).

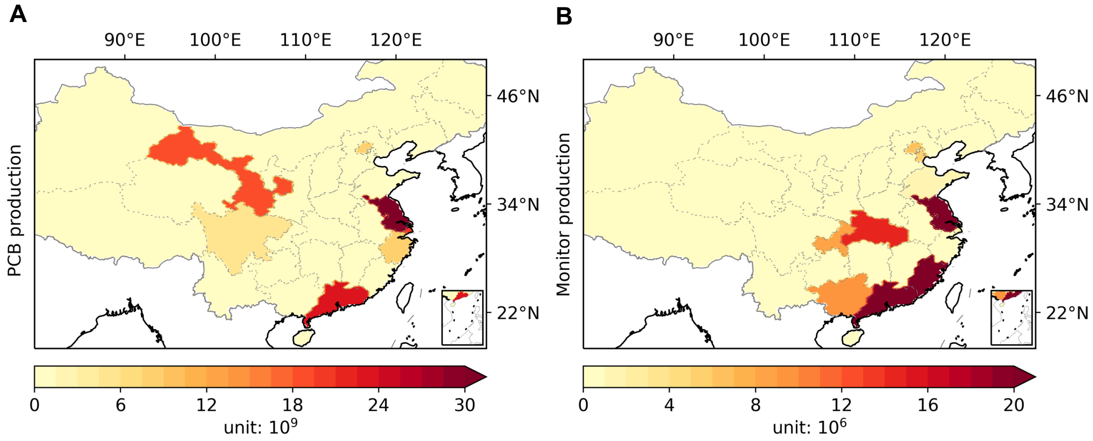

Fig. S9. Spatial distributions of integrated circuit plate production and screen monitor production in China by province. Panel (A) shows the integrated circuit plate production and (B) screen monitor production in each province. The values presented in the plots are the average of the available annual data during 2011-2020 from the China Industry Statistical Yearbook (2) (2011-2020 for integrated circuit plate production but we do not have data for 2017-2018; and 2011-2014 for screen monitor production). Although the definition of "screen monitor" here may not be identical to that of flat-panel display, and the "integrated circuit plate industry" is not necessarily equivalent to the semiconductor industry, we assume the screen monitor production and integrated circuit plate production to be reasonable approximations for the flat-panel display and semiconductor industries, respectively.

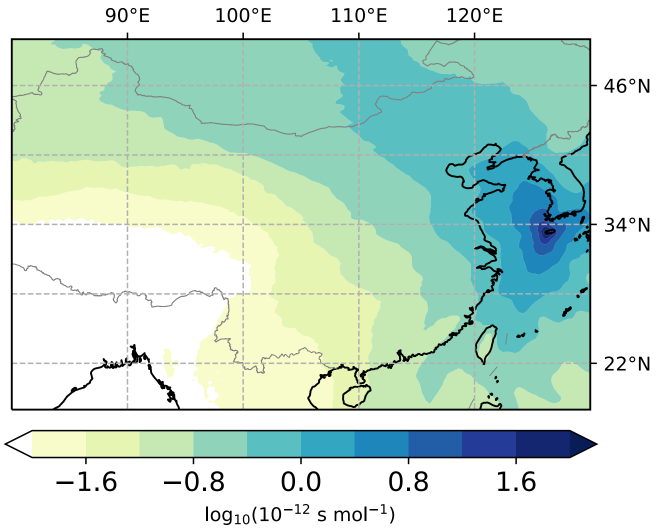

Fig. S10. Sensitivities of the measurements from Gosan (GSN) site to emissions in Eastern Asia. This plot shows the average sensitivity of the potential observations from Gosan in each hour of 2020 to emissions in space. The footprints from Gosan show relatively good coverage for emissions in the eastern regions of China, but provide less coverage in the western regions compared to the sites from the Chinese network used in this study (Figs. S14-15).

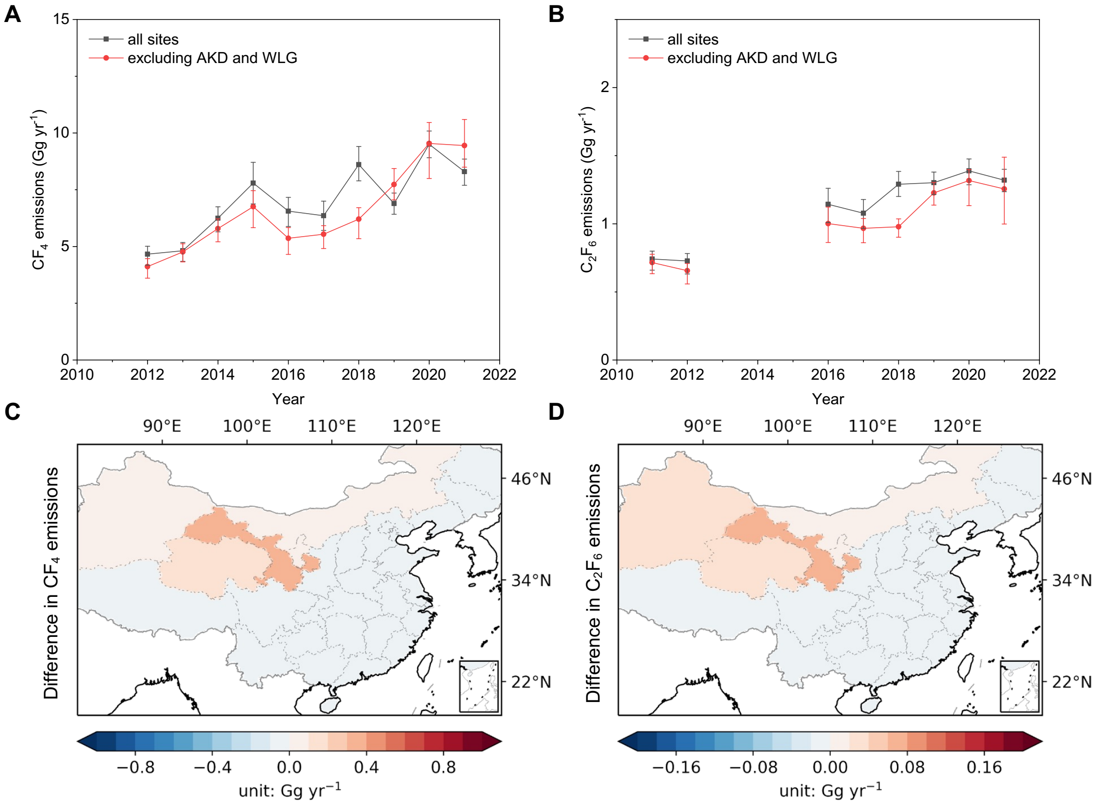

Fig. S11. Comparison between an inversion using measurements from all sites and an inversion excluding measurements from sites in the northwest of China (AKD and WLG). The left panels show the comparison for CF_4, and the right panels are for C_2F_6. The upper panels show the comparison of the derived emissions quantities in China, and the lower panel show the difference in emission spatial distributions between the two inversions (“inversion with all sites” minus “inversion excluding northwest”) averaged over the study period.

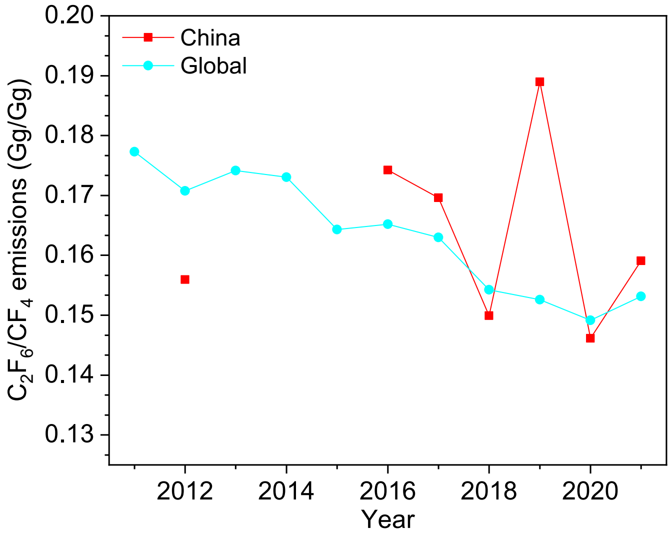

Fig. S12. C_2F_6/CF_4 emission ratios in China and globally. The global emission ratios exhibit a notable decreasing trend (slope of linear regression is $-0.00283\pm3.03\times10^{-4}$, $R^2=0.9$, $p<0.001$). The emission ratios in China do not display a significant decreasing trend ($p>0.05$).

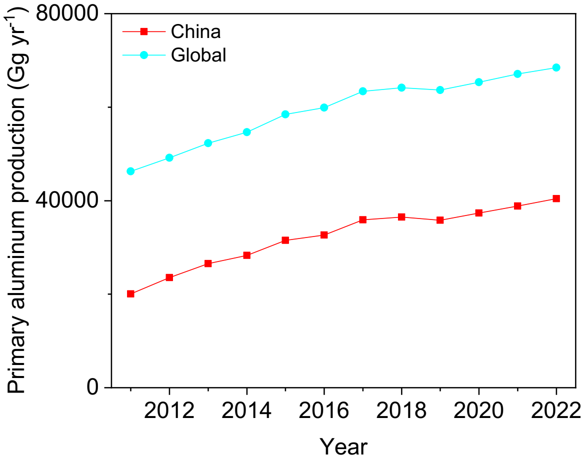

Fig. S13. Primary aluminum productions in China and global totals. The values in this plot are obtained from the International Aluminium Institute (3). The aluminum production for China in this plot is close to the sum of provincial aluminum production in Fig. S8, which were obtained from the Yearbook of Nonferrous Metals Industry of China (1), and the differences between the two data sources for aluminum production in China are ~10% over 2011-2013 and <2% over 2014-2019.

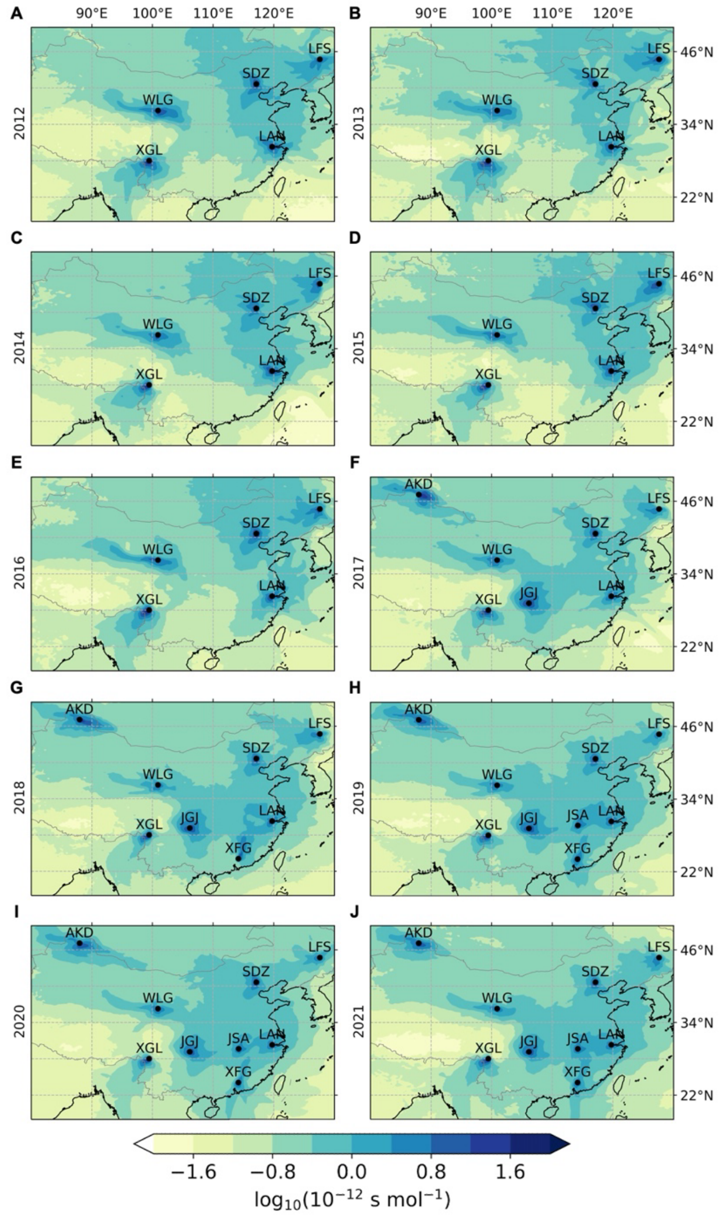

Fig. S14. Sensitivities of the measurements from nine sites to CF_4 emissions (footprints) in Eastern Asia with focus on China. Each plot of (A-J) shows the average value of mean footprints from all sites. The footprints provide good coverage for emissions in most regions of China and have small inter-annual variation.

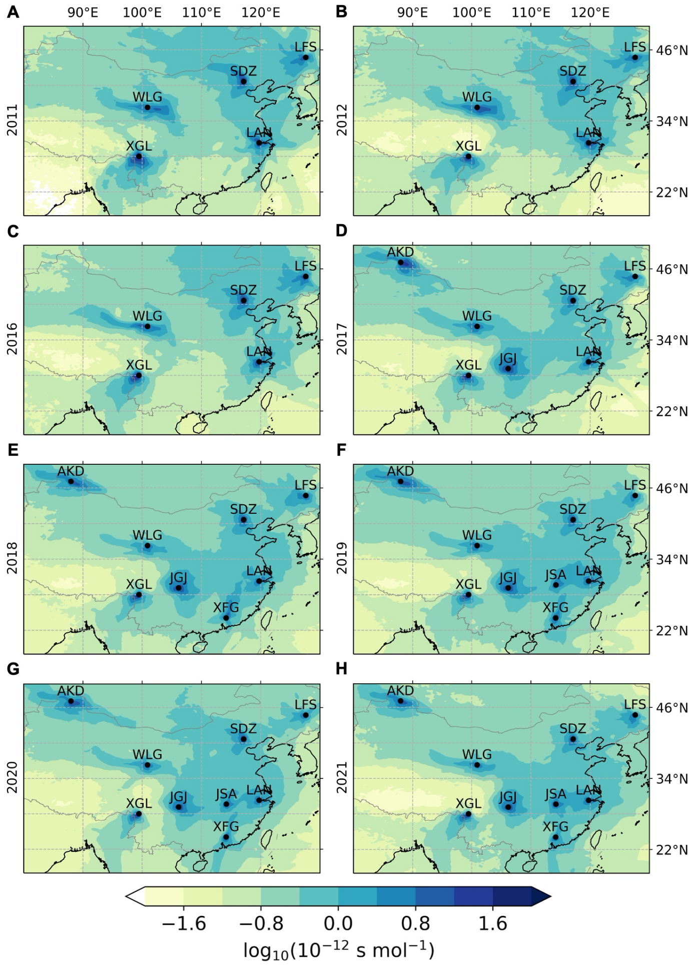

Fig. S15. Sensitivities of the measurements from nine sites to C_2F_6 emissions in Eastern Asia with focus on China. Each plot of (A-H) shows the average value of mean footprints from all sites. The footprints provide good coverage for emissions in most regions of China and have small inter-annual variations.

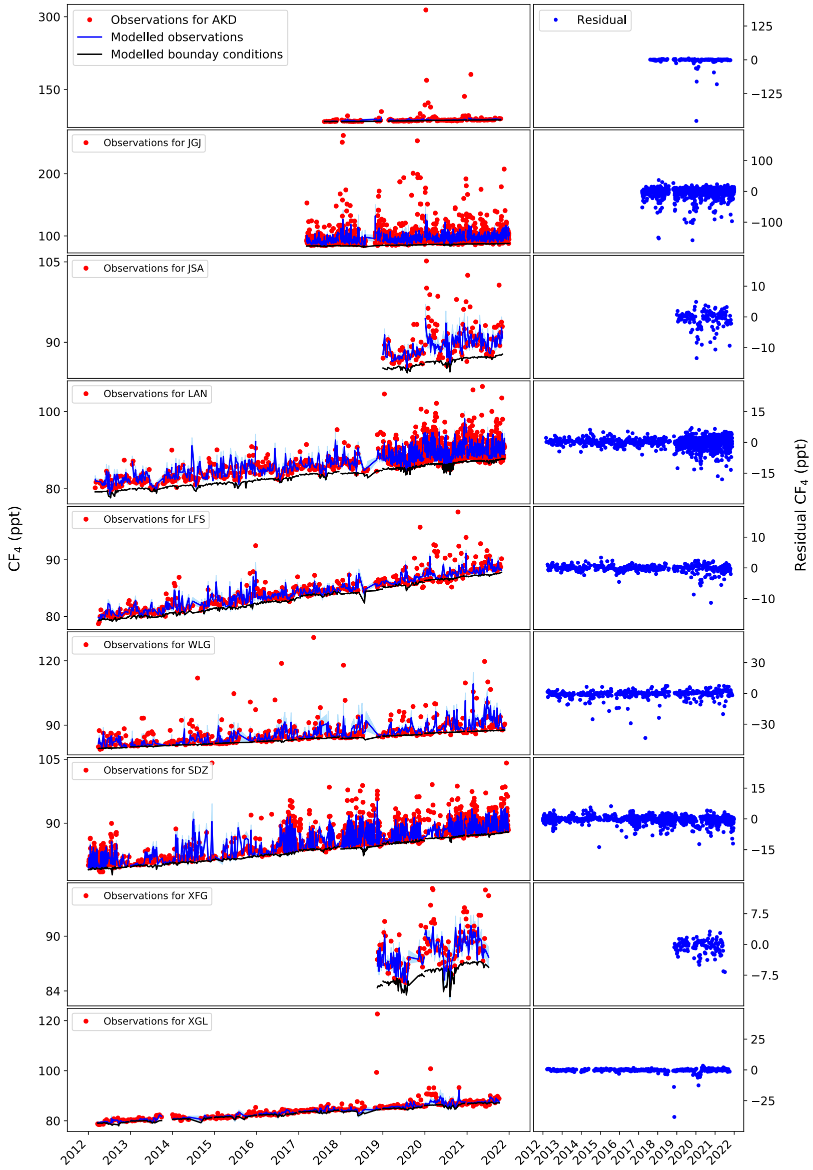

Fig. S16. Observed and simulated mole fractions of CF_4 for each site. In the left panels, the real observations used to estimate CF_4 emissions in this study (red dots, after re-sampling) are compared to the mole fractions modeled using the derived emissions (blue lines, with the shading representing for uncertainties). The black lines are the simulated a posteriori background mole fractions. The right panels show the differences between the observed and simulated values of the corresponding left panels.

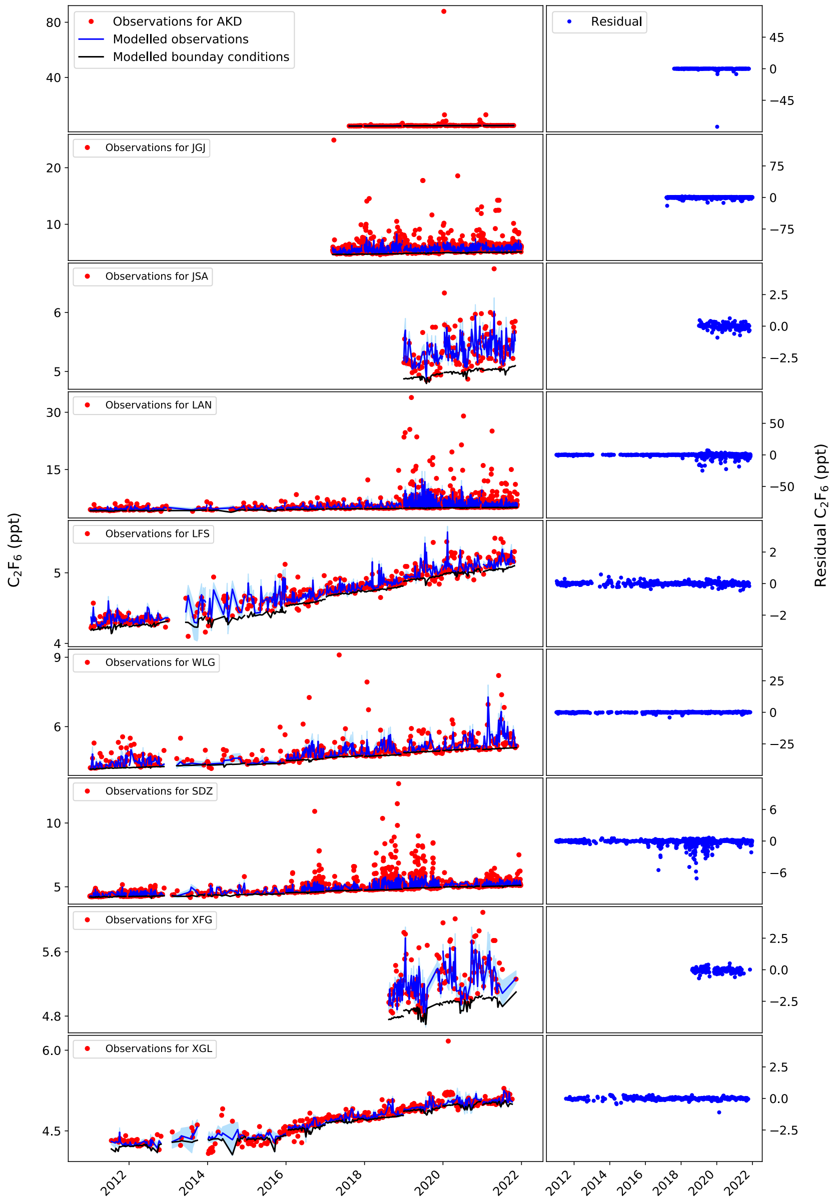

Fig. S17. Observed and simulated mole fractions of C_2F_6 for each site. As seen in Fig. S16 but for C_2F_6. The number of available measurements for C_2F_6 in 2013-2015 is smaller than the number of basis functions (the basic spatial unit in the inversion), which makes it hard to constrain the emissions and fit the observations. Thus, the emissions of C_2F_6 in 2013-2015 are not discussed further in the main text.

Table S1. Emissions in China and global emissions[a]

Year	Emissions in China ($Gg\ yr^{-1}$)		Global emissions[b] ($Gg\ yr^{-1}$)		China/global emission ratios[c] (%)	
	CF_4	C_2F_6	CF_4	C_2F_6	CF_4	C_2F_6
2011	/	0.74 (0.66-0.80)	11.0 (10.1-11.8)	1.94 (1.80-2.09)	/	38 (33-42)
2012	4.7 (4.2-5.0)	0.73 (0.63-0.78)	11.2 (10.3-12.0)	1.91 (1.77-2.04)	42 (36-46)	38 (32-42)
2013	4.8 (4.3-5.2)	/	11.1 (10.3-11.9)	1.94 (1.80-2.07)	44 (38-48)	/
2014	6.2 (5.6-6.7)	/	11.3 (10.4-12.1)	1.95 (1.82-2.07)	56 (49-61)	/
2015	7.8 (6.9-8.7)	/	12.0 (11.1-12.9)	1.97 (1.83-2.10)	65 (55-74)	/
2016	6.6 (5.9-7.2)	1.14 (1.01-1.26)	12.9 (12.1-13.8)	2.14 (2.00-2.28)	51 (44-56)	54 (46-60)
2017	6.4 (5.7-7.0)	1.08 (0.97-1.18)	13.9 (13.0-14.8)	2.27 (2.13-2.41)	46 (40-51)	48 (42-53)
2018	8.6 (7.9-9.4)	1.29 (1.20-1.39)	14.3 (13.4-15.2)	2.21 (2.07-2.35)	60 (54-67)	59 (53-64)
2019	6.9 (6.4-7.4)	1.30 (1.21-1.38)	14.4 (13.5-15.4)	2.20 (2.07-2.34)	48 (43-52)	59 (53-64)
2020	9.5 (8.9-10.1)	1.39 (1.29-1.48)	14.5 (13.5-15.6)	2.17 (2.03-2.31)	66 (59-71)	64 (58-70)
2021	8.3 (7.7-8.9)	1.32 (1.24-1.40)	/	/	/	/

(a) All the uncertainties are the 68% uncertainty intervals (or 1-sigma uncertainties).
(b) The global emissions were obtained from Laube and Tegtmeier (4) from AGAGE data.
(c) The China/global emission ratios were calculated by dividing emissions in China by global emissions. Emissions in China were obtained from the Markov chain from the inversion. Global emissions were assumed to follow normal distributions (parameterized by the means and standard deviations) and sampled by a Markov chain Monte Carlo method.

Table S2. Aluminum production (Gg yr^{-1}) in each subregion in each year[a]

Year	North	Northeast	East	Central	South	Southwest	Northwest
2011	2823	212	2287	4628	651	2782	4753
2012	2925	263	2298	4447	665	3073	6579
2013	3288	305	2519	4011	648	3627	8758
2014	3290	244	6147	3859	570	3187	11019
2015	3350	459	8201	3316	576	3377	12228
2016	4285	443	8395	3035	784	3115	12641
2017	4712	492	8808	2941	1206	3626	13404
2018	5358	570	9165	2563	1865	3755	13172
2019	5994	724	8202	1899	2278	4224	12653

(a) The aluminum production was obtained from the Yearbook of Nonferrous Metals Industry of China (1). The definition of the subregions can be found in Fig. S3.

Table S3. Period and frequency of the flask sampling and in situ measurement at each site

Station	Short name	Sampling frequency	CF_4 measurement periods	C_2F_6 measurement periods
Shangdianzi	SDZ	In situ/2h	2011-2012, 2016-2021[a]	2011-2012, 2016-2021[a]
		Weekly flasks	2012-2021	2011-2021
Mt. Waliguan	WLG	Weekly flasks	2012-2021	2011-2021
Lin'an	LAN	Weekly flasks	2012-2018	2011-2018
		Daily flasks	2019-2021	2019-2021
Longfengshan	LFS	Weekly flasks	2012-2021	2011-2021
Shangri-La	XGL	Weekly flasks	2012-2021	2011-2021
Jiangjin	JGJ	Daily flasks	2017-2021	2017-2021
Akedala	AKD	Weekly flasks	2017-2021	2017-2021
Jinsha	JSA	Weekly flasks	2019-2021	2019-2021
Xinfeng	XFG	Weekly flasks	2018-2021	2018-2021

(a) The lack of in situ data at SDZ during 2013-2015 was due to a malfunction of the instrument.

Dataset S1 (separate file). Derived CF_4 and C_2F_6 emissions in China (not including emissions from Hong Kong, Macao, Taiwan, and ocean regions).

Dataset S2 (separate file). Observations of CF_4 mole fractions used to derived CF_4 emissions in China and the subregions.

Dataset S3 (separate file). Observations of C_2F_6 mole fractions used to derived C_2F_6 emissions in China and the subregions.

SI References

1. China Nonferrous Metals Industry Association, *The Yearbook of Nonferrous Metals Industry of China (in Chinese)* (China Nonferrous Metals Industry Yearbook Press, Beijing, 2020).

2. Department of Industry Statistics, National Bureau of Statistics of China, *China Industry Statistical Yearbook (in Chinese)* (China Statistics Press, Beijing, 2021).

3. International Aluminium Institute, Primary Aluminium Production. Available at: https://international-aluminium.org/statistics/primary-aluminium-production/ [Accessed 1 August 2023].

4. J. C. Laube, S. Tegtmeier, “Chapter 1: Update on Ozone-depleting Substances (ODSs) and Other Gases of Interest to the Montreal Protocol” in *Scientific Assessment of Ozone Depletion: 2022*, (World Meteorological Organization, 2022).
